# Impact of Family Environment in Rural China on Loneliness, Depression, and Internet Addiction Among Children and Adolescents

**DOI:** 10.3390/ejihpe15050068

**Published:** 2025-05-01

**Authors:** Yixiang Zhou, Meng Zheng, Yujie He, Jianghui Zhang, Tingting Guo, Qing Wang, Wen Chen

**Affiliations:** 1 School of Public Health, Sun Yat-sen University, Guangzhou 510080, China; zhouyx99@mail2.sysu.edu.cn (Y.Z.); zhengm29@mail2.sysu.edu.cn (M.Z.); heyj77@mail2.sysu.edu.cn (Y.H.); zhangjh379@mail2.sysu.edu.cn (J.Z.); guott6@mail2.sysu.edu.cn (T.G.); 2 Center for Migrant Health Policy, Sun Yat-sen University, Zhongshan Road 2, Guangzhou 510080, China

**Keywords:** rural China, child and adolescent, mental health, parenting styles, family environment

## Abstract

This study investigates the impact of family environments on the psychological well-being of children and adolescents in rural China, focusing on loneliness, depression, and internet addiction. Using a cross-sectional design, data were collected from 652 families in rural Hunan province to examine the role of family communication, caregiver psychological states, and parenting styles. Structural equation modeling revealed that family economic conditions influenced children’s mental health through primary caregivers’ emotional well-being, hostile parenting styles, and family communication. Notably, hostile parenting and poor family communication were the strongest mediators across all three psychological outcomes, consistently exhibiting significant associations with loneliness, depression, and internet addiction. The findings highlight the crucial role of parenting styles and poor family communication in shaping children’s psychological health, emphasizing the need for interventions that enhance family communication and promote supportive parenting. Addressing caregiver emotional well-being and adjusting parental expectations may serve as effective strategies for improving mental health outcomes among rural youth.

## 1. Introduction

By the end of 2022, the overall urbanization rate in China reached 65%, with the acceleration of urbanization leading to large-scale rural population migration to cities (People’s Daily Overseas Edition, 2023). In 2023 alone, more than 130 million people from rural areas moved to cities to work, over half of whom were parents within rural families (National Bureau of Statistics of China, 2024). This trend in population movement has caused significant changes in the structure of rural families, particularly reflecting in the rise of children left and older adults in rural areas (Song et al., 2024). According to statistics from relevant Chinese agencies, in 2020, there were 108 million children nationwide, an increase of 30.46 million from 2010, who could not live with both parents, including 41.77 million rural left-behind children (National Bureau of Statistics of China, 2023). Correspondingly, the left-behind elderly people have become the main caregivers of the left-behind children (M. Zhao et al., 2021). This transformation in family structure not only reshapes the family living environment in rural areas but also has profound effects on the psychological health of left-behind children and adolescents (Ye & Lu, 2011; C. Zhao et al., 2018). Studies have shown that compared to their urban peers, these left-behind children and adolescents are more prone to experiencing mental disorders (N. Chen et al., 2019), including loneliness (X. Chen et al., 2014) and depression (Mao & Zhao, 2012).

In addition, internet addiction due to the lack of direct care and supervision from parents has become an urgent mental health issue among left-behind children and adolescents (D. Wu et al., 2024). As the economic situation improves and new rural construction advances, rural areas are increasingly equating with urban areas in terms of network access and information technology use (Leng, 2022). Notably, by 2020, rural areas had surpassed urban regions in mobile phone usage rates (Xinhua News Agency, 2022). However, the rural population primarily engages in entertainment activities online, with less emphasis on using digital tools for educational or developmental purposes compared to urban users (Southern Metropolis Daily, 2022). This discrepancy poses potential risks for internet addiction among rural youth, making it crucial to include this behavior alongside loneliness and depression in studies exploring potential influencing factors.

Previous research has identified multiple factors that may influence mental and behavioral issues during childhood and adolescence (Fellmeth et al., 2018), including biological (De Figueiredo et al., 2021), behavioral (Xiao et al., 2021), physical health (Linardon et al., 2023), and family environmental factors (Glynn et al., 2021). Given the primacy of the family as the first arena of socialization during childhood and adolescence, and influenced by China’s family-centric traditional values, the impact of the family environment is likely to be magnified (Wei & Chen, 2014). Although there is no unified and clear definition of the concept of family environment in academia (J. Lee et al., 2020), it is generally believed to cover multiple dimensions such as the relationship between family members, communication patterns, and emotional support (Hohashi & Honda, 2011; Xie et al., 2022). These dimensions not only reflect the material living conditions and social status of the family but also include the interaction between family members and their overall language, behavior, and emotions. Previous evidence suggested various family environmental factors are believed to be likely to cause psychological and behavioral problems in children and adolescents (Meherali et al., 2021), such as family economic situation (Allen et al., 2014), parental psychological problems (Bennett et al., 2012), and parenting methods (Russell et al., 2020). Given the current transformations in rural family structures exploring how the family environment affects the mental health of left-behind children not only aids in better understanding how various components within the family environment contribute to issues such as loneliness, depression, and internet addiction but also is vital for developing effective intervention strategies to support this vulnerable group (Kenney & Chanlongbutra, 2020; F. Zhao & Yu, 2016).

**Figure 1:**
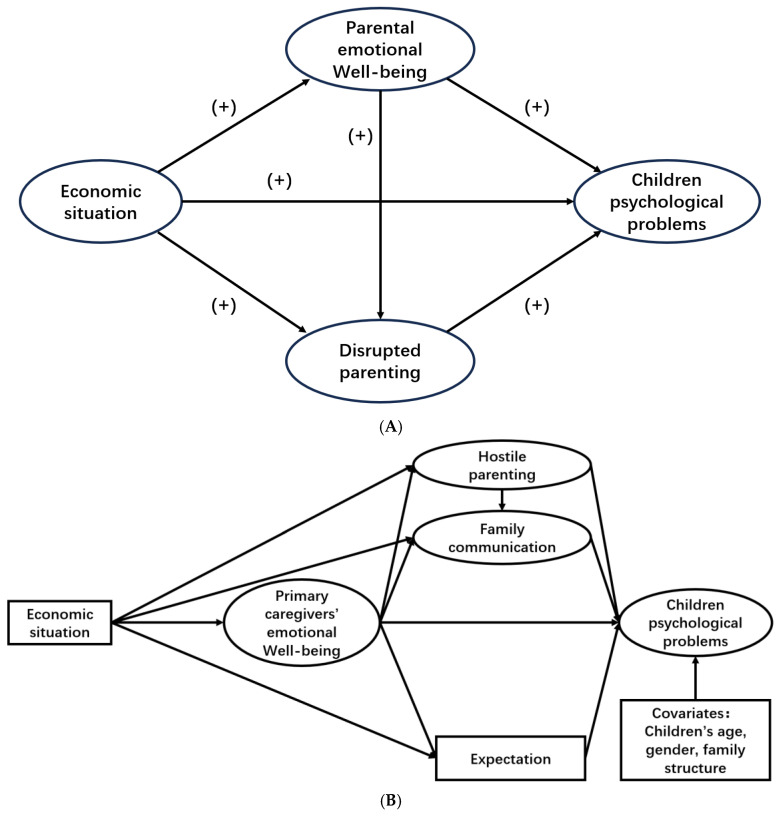
(**A**). The Original Family Stress Model framework. Note. “(+)” denotes a positive relationship between variables. (**B**). The Modified Family Stress Model framework.

Many previous studies have discussed how different family environmental factors affect the psychological and behavioral problems of children and adolescents (Fan & Chen, 2012; Torche et al., 2024; Zhou et al., 2021). The family stress model (FSM) is a theoretical framework commonly used in previous studies to analyze how economic factors in the family affect the psychological state of parents and then influence the mental and behavioral health of their children (Conger et al., 1994; Mistry et al., 2002). The core components of the original FSM, shown in Figure 1A, include family economic conditions, parental psychological distress, parenting style, and children’s psychological and behavioral outcomes (Conger et al., 1994). It was initially applied to families in rural areas in the Midwest of the United States and was later extended to various populations (Barajas-Gonzalez & Brooks-Gunn, 2014; Mistry et al., 2002), demonstrating good reliability, validity, and stability (Barajas-Gonzalez & Brooks-Gunn, 2014). However, the original model does not reflect the impact of family communication and parental expectations, which have been shown to play a critical role in the psychological and behavioral development of children and adolescents in rural areas of China (Prime et al., 2020; L. Zhang, 2024). Therefore, as shown in Figure 1B, we incorporated these two factors—family communication and parental expectations—into the original FSM. The resulting modified FSM was used in this study to examine the comprehensive relationships between multiple family environmental factors and children and adolescents’ mental health, including loneliness, depression, and internet addiction.

## 2. Materials and Methods

### 2.1. Research Design and Study Participants

From September 2023 to May 2024, this study adopted a cross-sectional research design, and multi-stage random sampling was used to select the study participants. Hunan Province, located in southern China, was selected as the study site due to its status as a major agricultural province with a high proportion of population outmigration, resulting in a substantial number of left-behind children. This context provides an appropriate setting for examining the effects of family environment on the mental health of children and adolescents in rural areas. All fourteen cities in Hunan province were divided into three levels according to their economic development status. In each level, a representative city was randomly selected. Two towns were randomly selected in each selected city, and then two villages were randomly selected in each town as specific survey sites. In each village, we recruited children and adolescents aged 9–18 years old and their main caregivers to participate in the face-to-face survey. Exclusion criteria included: (1) either the child or the primary caregiver having a diagnosed severe mental illness (e.g., schizophrenia, bipolar disorder); (2) the child having a diagnosed neurodevelopmental disorder (e.g., autism spectrum disorder, intellectual disability); and (3) failure to complete all questionnaires independently. All participating children and adolescents, along with their primary caregivers, provided written informed consent prior to the survey, and the research plan was approved by the Ethics Committee of the School of Public Health of Sun Yat-sen University (approval number: IRB-SYSU-2024-001).

#### Sample

**Figure 2:**
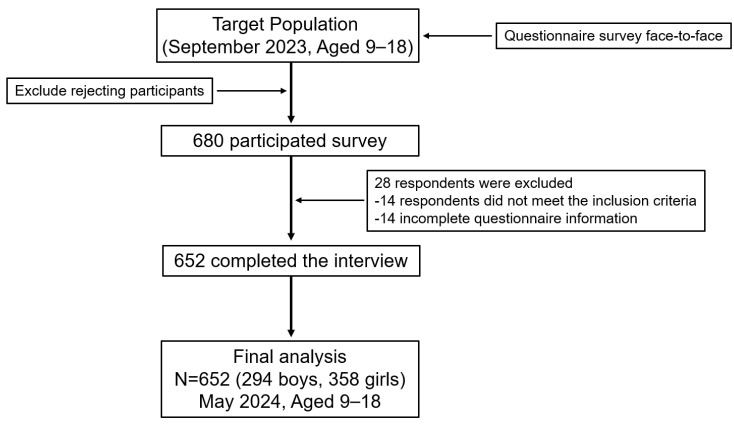
Flowchart of population included in final analysis (*n* = 652).

Figure 2 presents a flow chart of the study sample selection process. Initially, 680 families were recruited for participation. After excluding 14 families due to incomplete key information and another 14 families who did not meet the inclusion and exclusion criteria, a total of 652 families were included in the final analysis. Given that this study was conducted at the family level, when multiple children or adolescents from the same family met the inclusion criteria, one participant was randomly selected to complete the survey.

Among the 652 children and adolescents included, 297 (45.6%) were classified as children (aged 6–12 years), and 355 (54.4%) as adolescents (aged 12–18 years). The average age of the participants was 13.06 years (SD = 2.16), and 45.1% (*n* = 294) were male. In terms of primary caregiving arrangements, 259 participants (39.7%) resided with their grandparents (either maternal or paternal), whose ages ranged from 56 to 71 years (M = 62.1, SD = 6.9). This proportion was slightly higher than those cared for by both parents (32.1%) or by a single parent (28.2%).

### 2.2. Measures

This study employed several validated scales to measure loneliness, depression, and internet addiction among adolescents in rural areas, as well as a structured assessment to evaluate the family environment. The family environment was assessed through a combination of responses provided by both adolescents and their primary caregivers, allowing for a comprehensive understanding of the family environment.

#### 2.2.1. Loneliness Among Children and Adolescents

The simplified UCLA Loneliness Scale (R-UCLA) was utilized to assess loneliness (C. Wang et al., 2024). This scale includes three questions probing the frequency of feeling a lack of companionship, feeling left out, and feeling isolated, based on experiences within the past year. Responses are scored on a 0–3 scale, with the total score ranging from 0 to 9. A higher total score indicates a higher level of loneliness, reflecting a lower sense of social connection. Previous studies have demonstrated good reliability and validity of the R-UCLA when used among Chinese populations, confirming its cultural adaptability (Cronbach’s alpha coefficient: 0.91) (Yuan et al., 2024). In this study, the scale also demonstrated high internal consistency (Cronbach’s alpha = 0.92).

#### 2.2.2. Depression Among Children and Adolescents

Depression was evaluated using an eight-item version of the Center for Epidemiologic Studies Depression Scale (CES-D) (S. Liu et al., 2023). This tool assesses depressive symptoms based on experiences over the past week, with one item related to loneliness excluded to avoid overlapping with the loneliness scale and to focus more specifically on other depressive symptoms, yielding a potential total score range of 0 to 21 (S. L. Lee et al., 2021). In this study, a four-point scale was used (0 = not at all or very rarely, 1 = rarely, 2 = often, 3 = almost always), so that higher total scores indicate higher levels of depressive symptoms and lower emotional well-being. Previous studies have demonstrated that the CES-D has excellent reliability and validity when used among Chinese populations, confirming its cultural applicability (Gou et al., 2022). The internal consistency of this scale in the current study was high (Cronbach’s alpha = 0.957).

#### 2.2.3. Internet Addiction Among Children and Adolescents

The Revised Internet Addiction Scale (CIAS-R) was utilized to assess internet addiction (Y. Wang et al., 2024). It comprises 26 items that evaluate aspects such as the frequency and duration of internet use, impulse control, daily life disruption, and emotional experiences during internet use, based on the past year’s experiences. The scale employs a four-point Likert scale, ranging from 1 (completely inconsistent with my experience) to 4 (completely consistent with my experience). The minimum and maximum scores for the CIAS-R are 26 and 104, respectively. A higher total score indicates a greater likelihood of internet addiction. Previous studies have shown that the CIAS-R demonstrates excellent reliability and validity when applied to Chinese populations, confirming its suitability for assessing internet addiction within this cultural context (Mak et al., 2014). The internal consistency for this scale in the current study was high (Cronbach’s alpha = 0.975).

#### 2.2.4. Family Environment: The Family Environment Was Comprehensively Assessed Through Five Sub-Dimensions (X. Ding et al., 2024; Yang & Zhao, 2020; Young & Hannum, 2018)

##### Economic Situation

Assessed based on the overall income of the household. The economic situation of the surveyed families was categorized into five levels based on the rural per capita monthly income in Hunan Province (approximately 2000 CNY), divided by percentiles into the following categories: below 1000, 1000–2000, 2000–3000, 3000–5000, and above 5000.

##### Parenting Styles

Evaluated through two scales measuring warmth and hostility. The warmth sub-scale, derived from the Child Rearing Questionnaire, was used to assess warm parenting, focusing on positive engagement in child-rearing (Paterson & Sanson, 1999). This sub-scale included five items indicating positive emotional expressions by primary caregivers toward their children, each scored on a 5-point scale (1 = none of the time to 5 = all the time), with an internal consistency of 0.917. The hostility sub-scale, derived from the Early Childhood Longitudinal Study of Children (T. Liu et al., 2024), was used to assess hostile parenting, evaluating negative expressions and behaviors toward children. This sub-scale also included five items, scored from 1 (rarely) to 5 (almost always), with an internal consistency of 0.909.

##### Family Communication

Family communication was assessed using the Expressiveness subscale from the Chinese version of the Family Environment Scale (FES-CV), which specifically reflects the extent to which family members are encouraged to openly express their emotions and opinions. The original FES-CV consists of 90 items across 10 subscales. However, based on previous validation studies among Chinese adolescents, two subscales—Independence and Moral-Religious Emphasis—were excluded due to poor internal consistency and limited cultural relevance in mainland China (D. Liu et al., 2008). As a result, the revised version of the FES-CV used in this study contains 72 items across the remaining eight subscales: cohesion, expressiveness, conflict, achievement orientation, intellectual-cultural orientation, active-recreational orientation, organization, and control. In this study, only the Expressiveness subscale was used to evaluate family communication, and its internal consistency was acceptable (Cronbach’s alpha = 0.793).

##### Caregivers’ Emotional Well-Being

The Caregivers’ Emotional Well-being was comprehensively assessed through the evaluation of loneliness and depression, which were measured using the R-UCLA scale (L.-J. Liu & Guo, 2007) and the CES-D scale (Y. Ding et al., 2022), respectively.

##### Caregivers’ Expectations for Children

This dimension assesses the expectations that primary caregivers have for their children. Expectations were evaluated through two specific questions designed to capture the educational and occupational aspirations of caregivers for their children. The questions included: (1) “What level of education do you expect your child to achieve?” with response options ranging from primary school to master’s degree or above, and (2) “What kind of career do you envision for your child?” with response options covering various career types from agricultural work to professional careers (Li et al., 2017).

Responses were classified into three levels—low, medium, and high—based on the specificity and ambition of the caregivers’ aspirations. For educational expectations, low expectations included aspirations for completing primary or junior high school, medium expectations included completing high school or university, while high expectations corresponded to aspirations for obtaining a master’s degree or higher. For occupational expectations, low expectations referred to agricultural or household work, medium expectations included technical jobs or positions in the public sector, and high expectations included professional careers or management positions.

To derive an overall expectation level, we combined educational and occupational expectations using a scoring approach. Each response was assigned a score (low = 1, medium = 2, high = 3), and the scores for educational and occupational expectations were summed to obtain a total score ranging from 2 to 6. The overall classification was as follows: total scores of 2 or 3 were classified as low expectations, a total score of 4 was classified as medium expectations, and total scores of 5 or 6 were classified as high expectations (Yang & Wang, 2025).

### 2.3. Covariates

In this study, we included both primary caregivers’ and children’s demographics as covariates. Children’s demographics included sex, grade, and caregiving patterns (1 = both parents caregiving, 2 = one parent caregiving, 3 = cross-generational caregiving). Primary caregivers’ demographics included educational backgrounds (1 = primary school or below, 2 = junior high school, 3 = high school, 4 = university, 5 = master’s degree or above).

### 2.4. Statistical Analysis

The sociodemographic characteristics of the participants were described using means and standard deviations (SD) for continuous variables, and frequencies and proportions for categorical variables. Subsequently, Cronbach’s alpha coefficients were used to assess the internal consistency of each scale employed in the study. Correlation analyses were conducted to describe the relationships among the five indicators of the family environment, as well as between these indicators and mental health outcomes (loneliness, depression, and internet addiction) in children and adolescents. Warm parenting was excluded from the model due to its non-significant associations with economic situation and the outcomes of loneliness, depression, and internet addiction. Accordingly, all paths associated with warm parenting were removed in the subsequent structural equation modeling analysis.

**Figure A1:**
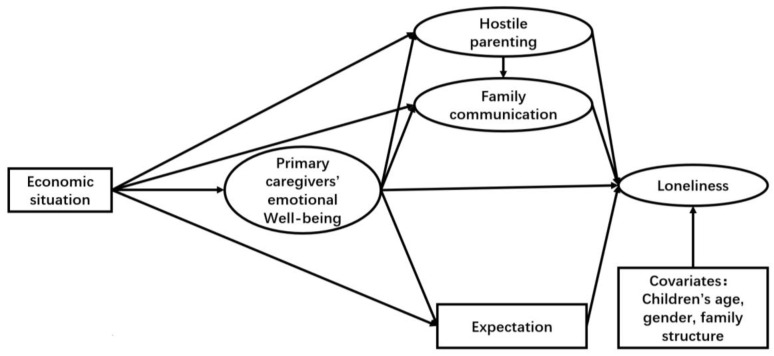
The Modified Family Stress Model framework for loneliness.

**Figure A2:**
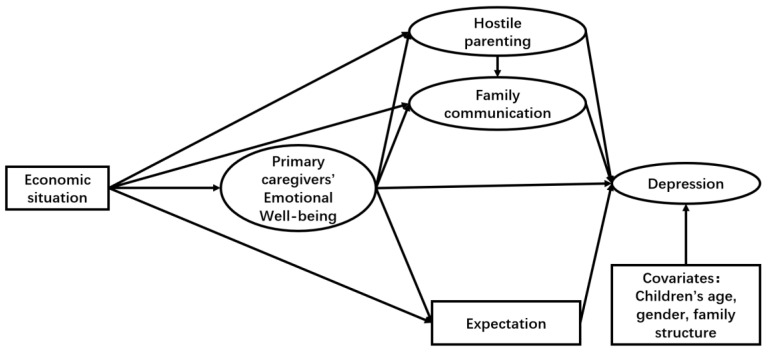
The Modified Family Stress Model framework for depression.

**Figure A3:**
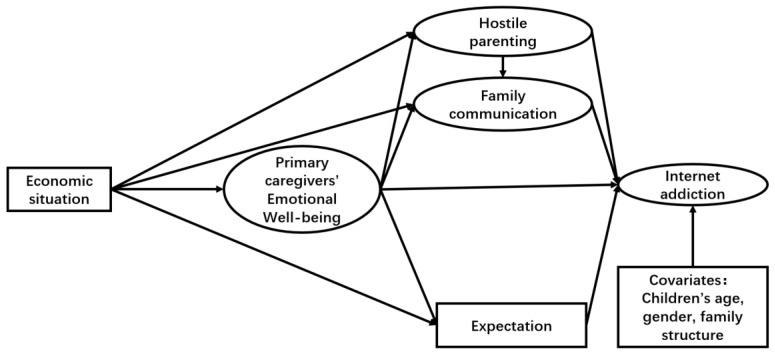
The Modified Family Stress Model framework for internet addiction.

The identified correlated variables were analyzed using structural equation modeling (SEM) based on the modified Family Stress Model (FSM) framework (Figure A1, Figure A2 and Figure A3). The robustness of the estimated parameters was tested using the bootstrap method with 5000 resamples. Due to the non-normality in the data, we employed SEM with robust maximum-likelihood estimation to test the complex relationships between the study variables in both the original and modified FSM. Specifically, bootstrapping (with 5000 resamples) was used to assess the indirect effects of economic situation, parenting style, family communication, primary caregivers’ emotional well-being, and expectations on children’s psychological problems through pathways such as disrupted parenting, family communication, and primary caregivers’ emotional well-being. This approach allowed for the assessment of both direct and indirect effects, enhancing the robustness of the interval estimates. Furthermore, to explore the relationships between expectations and loneliness, depression, and internet addiction, the high expectation group and the low expectation group were compared separately with the medium expectation group using t-tests. The data-model fitting effect was assessed using the following indices: Comparative Fit Index (CFI) ≥ 0.90, Tucker–Lewis Index (TLI) ≥ 0.90, Standardized Root Mean Square Residual (SRMR) ≤ 0.06, Root Mean Square Error of Approximation (RMSEA) ≤ 0.08, and the normed chi-square (*χ*^2^*/d.f.*) ≤ 3.0. The applicability of the original FSM and the modified FSM in rural China was compared based on these fit indices.

## 3. Results

### 3.1. Sociodemographic Characteristics of the Participants

Descriptive statistics for psychological and behavioral outcome variables are summarized in Table 1. The mean score for depressive symptoms as measured by the CES-D was 9.40 (SD = 2.84). The mean score for loneliness, assessed using the Revised UCLA Loneliness Scale (R-UCLA), was 21.63 (SD = 6.63). The mean score for internet addiction, as assessed by the CIAS-R, was 79.65 (SD = 21.55).

### 3.2. Associations of Family Environment with Loneliness, Depression, and Internet Addiction in Children and Adolescents

The results of the correlation analysis (Table A1) showed that all variables involved in the structural equation modeling were correlated. Table 2 and Table 3 present the fit indices for the structural equation models under both the original FSM and modified FSM frameworks. Under the modified FSM framework, all three models (Figure A1, Figure A2 and Figure A3) had a normed chi-square (*χ*^2^*/d.f.*) below 3, CFI and TLI values above 0.95, and RMSEA and SRMR values below 0.05, indicating a good model fit. The overall fit of the structural equation models under the modified FSM framework was slightly better compared to the original FSM framework.

**Figure 3:**
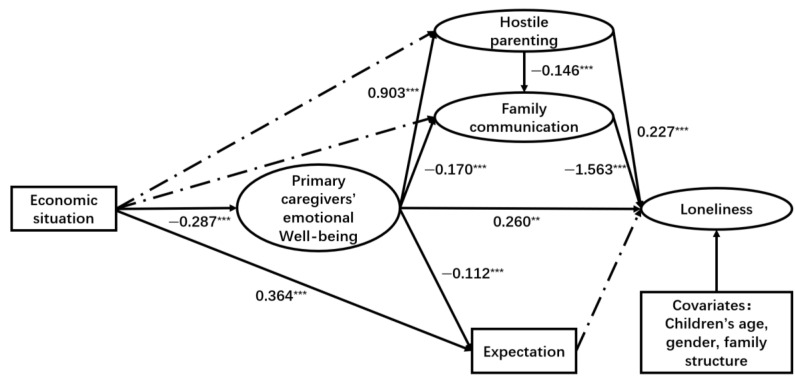
Results of SEM path analysis (loneliness). Note. ** *p* < 0.01, *** *p* < 0.001. Coefficients are unstandardized path coefficients. The solid lines represent significant paths (*p* < 0.05). The dashed lines represent non-significant paths. Children’s age, gender, family structure are included as covariates.

**Figure 4:**
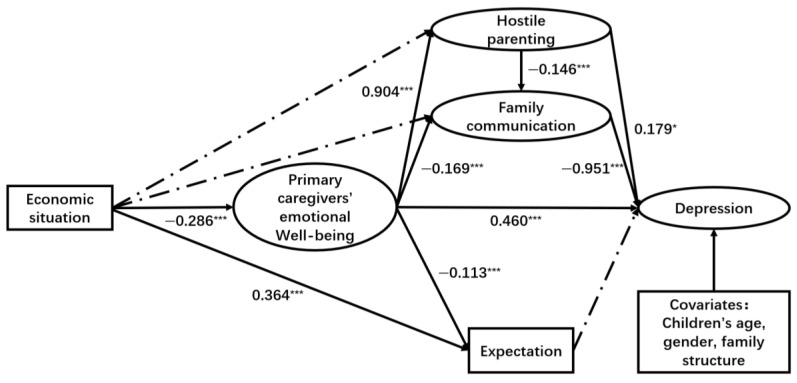
Results of SEM path analysis (depression). Note. * *p* < 0.05, *** *p* < 0.001. Coefficients are unstandardized path coefficients. The solid lines represent significant paths (*p* < 0.05). The dashed lines represent non-significant paths. Children’s age, gender, family structure are included as covariates.

**Figure 5:**
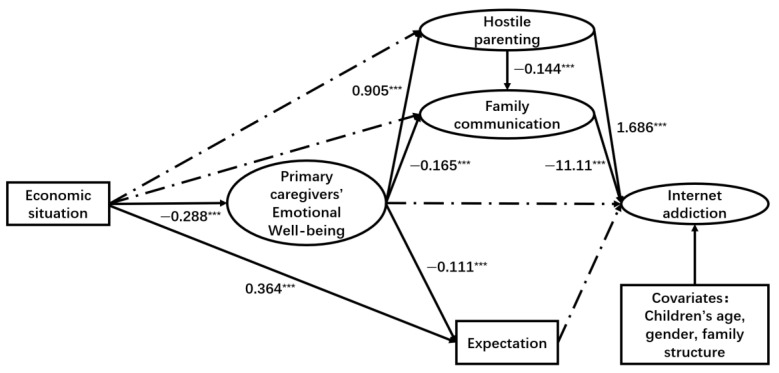
Results of SEM path analysis (internet addiction). Note. *** *p* < 0.001. Coefficients are unstandardized path coefficients. The solid lines represent significant paths (*p* < 0.05). The dashed lines represent non-significant paths. Children’s age, gender, family structure are included as covariates.

As illustrated in Figure 3, Figure 4 and Figure 5, both hostile parenting styles and family communication have direct and significant impacts on children’s loneliness (hostile parenting → loneliness: β = 0.227, *p* < 0.001; family communication → loneliness: β = −1.563, *p* < 0.001), depression (hostile parenting → depression: β = 0.179, *p* < 0.05; family communication → depression: β = −0.951, *p* < 0.001), and internet addiction (hostile parenting → internet addiction: β = 1.686, *p* < 0.001; family communication → internet addiction: β = −11.11, *p* < 0.001). Additionally, primary caregivers’ emotional well-being had significant positive effects on children’s loneliness (β = 0.260, *p* < 0.01) and depression (β = 0.460, *p* < 0.001). However, this significant association was not observed between primary caregivers’ emotional well-being and internet addiction.

The economic situation showed a significant negative relationship with primary caregivers’ emotional well-being (β = −0.287, *p* < 0.001 for loneliness model; β = −0.286, *p* < 0.001 for depression model; β = −0.288, *p* < 0.001 for internet addiction model) and expectation (β = 0.364, *p* < 0.001 for three models). Furthermore, primary caregivers’ emotional well-being had a significant positive association with hostile parenting (β = 0.903, *p* < 0.001 for loneliness model; β = 0.904, *p* < 0.001 for depression model; β = 0.905, *p* < 0.001 for internet addiction model), while it showed a significant negative association with family communication (β = −0.170, *p* < 0.001 for loneliness model; β = −0.169, *p* < 0.001 for depression model; β = −0.165, *p* < 0.001 for internet addiction model). Additionally, primary caregivers’ emotional well-being had a significant negative association with expectation (β = −0.112, *p* < 0.001 for loneliness model; β = −0.113, *p* < 0.001 for depression model; β = −0.111, *p* < 0.001 for internet addiction model).

However, caregivers’ expectations for their children did not show a direct significant influence on children’s loneliness (β = −0.016, *p* = 0.534), depression (β = −0.026, *p* = 0.349), or internet addiction (β = 0.265, *p* = 0.178). Additionally, there was no significant direct correlation between family economic situations and family communication across the three models. The study found that economic situation could indirectly influence children’s loneliness, depression, and internet addiction symptoms through primary caregivers’ emotional well-being, which in turn affects family communication and hostile parenting styles.

These indirect effects were validated using the bootstrap method, as shown in Table 3, Table 4 and Table 5. For loneliness, Path b (economic situation → primary caregivers’ emotional well-being → hostile parenting → family communication → child loneliness, β = −0.059, 95% CI: (−0.089, −0.030)), Path f (economic situation → primary caregivers’ emotional well-being → child loneliness, β = −0.075, 95% CI: (−0.121, −0.028)), Path g (economic situation → primary caregivers’ emotional well-being → hostile parenting → child loneliness, β = −0.059, 95% CI: (−0.100, −0.018)), and Path i (economic situation → parents’ emotional well-being → family communication → loneliness, β = −0.076, 95% CI: (−0.113, −0.040)) were significant.

Similarly, for depression, Path b (economic situation → primary caregivers’ emotional well-being → hostile parenting → family communication → child depression, β = −0.036, 95% CI: (−0.060, −0.012)), Path f (economic situation → primary caregivers’ emotional well-being → child depression, β = −0.132, 95% CI: (−0.184, −0.080)), Path g (economic situation → primary caregivers’ emotional well-being → hostile parenting → child depression, β = −0.046, 95% CI: (−0.087, −0.005)), and Path i (economic situation → parents’ emotional well-being → family communication → depression, β = −0.046, 95% CI: (−0.076, −0.016)) showed significance.

For internet addiction, only Path b (economic situation → primary caregivers’ emotional well-being → hostile parenting → family communication → internet addiction, β = −0.418, 95% CI: (−0.630, −0.206)), Path g (economic situation → primary caregivers’ emotional well-being → hostile parenting → internet addiction, β = −0.439, 95% CI: (−0.747, −0.132)), and Path i (economic situation → parents’ emotional well-being → family communication → internet addiction, β = −0.529, 95% CI: (−0.795, −0.262)) were significantly associated.

## 4. Discussion

The survey results indicate that 38.5%, 37.9%, and 5.8% of children and adolescents in rural Southern China exhibit symptoms of loneliness, depression, and internet addiction respectively. In comparison with the prevalence rates of loneliness (14.5%) and depression (25.2%) reported in China’s first “Rural Children’s Mental Health Survey”, the prevalence of loneliness and depression in this study is much higher, while the prevalence of internet addiction is lower than the 16.2% reported in the “2021 National Research on Internet Usage among Minors (China Foundation Development Forum Parallel Forum, 2022)”. These differences may be attributed to variations in regional samples, socio-cultural contexts, and levels of internet penetration. The lower prevalence of internet addiction in this study may be explained by limited internet access in rural areas and more stringent parental supervision over internet use, whereas the higher prevalence of loneliness and depression may be associated with the scarcity of mental health resources and highlight urban-rural disparities in mental health problems among children and adolescents (Chinese Communist Youth League, 2022). At the same time, our survey reveals that the prevalence rates of loneliness, depression, and internet addiction among left-behind children were 49.7%, 49.2%, and 8.6%, respectively, all much higher than those observed among non-left-behind children. Specifically, the prevalence of loneliness and depression among non-left-behind children were 14.8% and 13.9%, respectively, while no cases of internet addiction were reported in this group during our survey. These findings are consistent with the results of China’s first Rural Children’s Mental Health Survey Report released in 2022, which also indicated that left-behind children exhibited significantly higher rates of psychological and behavioral problems compared to their non-left-behind peers. These differences underscore the mental health vulnerability of left-behind children in rural areas. The substantially higher rates of psychological problems among this group may be attributed to prolonged parental absence, reduced emotional support, and weaker family bonds—factors that are closely tied to the structural changes in rural families brought about by large-scale migration (Ye & Lu, 2011; C. Zhao et al., 2018). This study further explored the impact of family environmental factors on loneliness, depression, and internet addiction among rural youths in China using structural equation modeling (SEM) within the modified Family Stress Model (FSM) framework. The results showed that the SEM under the modified FSM framework provided a slightly better fit than the SEM under the original FSM framework. Additionally, the analysis revealed that family economic status, the emotional well-being of primary caregivers, hostile parenting styles, and family communication had significant effects on the loneliness, depression, and internet addiction of rural children and adolescents. Specifically, caregivers’ emotional well-being was found to have a direct and significant impact on loneliness and depression, but no such direct association was observed with internet addiction. Furthermore, no significant pathways involving caregiver expectations were identified.

Previous research has demonstrated the significant influence of family economic situation on children’s and adolescents’ mental health problems, which aligns with our findings that the family economic situation indirectly influences loneliness, depression, and internet addiction among rural youths (Cui & Hong, 2021; J. Zhang, 2018). However, our study further revealed that these indirect effects are mainly achieved by influencing caregiver psychological states, which subsequently affect parenting styles and family communication.

In addition, our results indicated that hostile parenting styles and family communication were involved in all three significant pathways in the three SEM models (1. Economic situation → primary caregivers’ emotional well-being → hostile parenting → children’s psychological problems (loneliness, depression, internet addiction); 2. Economic situation → primary caregivers’ emotional well-being → hostile parenting → family communication → children’s psychological problems (loneliness, depression, internet addiction); 3. Economic situation → primary caregivers’ emotional well-being → family communication → children’s psychological problems (loneliness, depression, internet addiction)). However, in the internet addiction model, primary caregivers’ emotional well-being did not have a significant direct impact on internet addiction, suggesting that hostile parenting and poor family communication may have a stronger influence on loneliness, depression, and internet addiction compared to primary caregivers’ emotional well-being alone.

The significant involvement of hostile parenting styles and poor family communication across all three pathways underscores that poor caregiver psychological states, negative family communication, and hostile parenting are more likely to lead to loneliness and depression in children and adolescents. A recent study conducted in Taiwan on the relationship between children’s psychological issues and family and school involvement suggested that, influenced by traditional Chinese culture, children’s experiences in family and school play a more critical role in their psychological outcomes compared to those in other regions (Y. Ding et al., 2022). Therefore, considering previous studies, we believe that in the more traditional and family-centered rural areas of China, hostile parenting and negative family atmospheres not only directly impact the psychological state of children and adolescents but may also exacerbate these effects by weakening positive family interactions and communication, thereby increasing children’s rebellious sentiments (Suizzo & Cheng, 2007). In rural areas, where the family is the primary setting for socialization, family harmony and support are especially crucial for children’s psychological development. This indicates that improving family relationships and enhancing positive communication and emotional connections between parents and children are essential for preventing and alleviating loneliness and depression among children and adolescents (Tang et al., 2023).

Additionally, the lack of a significant direct impact of primary caregivers’ emotional Well-being on internet addiction in the internet addiction model suggests the unique characteristics of internet addiction among children and adolescents in rural areas. Specifically, internet addiction in rural youth may be more influenced by family atmosphere and parenting styles. Based on our findings and previous research, while psychological interventions for caregivers are important, direct interventions have certain challenges and limitations (H. Xu, 2019). Thus, improving family communication methods and optimizing parenting styles are more feasible and effective strategies. Such approaches not only mitigate the challenges of direct interventions due to primary caregivers’ emotional well-being but also effectively address issues of loneliness, depression, and internet addiction.

This study has several limitations. Firstly, the sample selection was confined to rural areas of a single province in China. Given China’s vast geographical range and regional variations in lifestyle and cultural backgrounds, the representativeness of the sample may be limited. This could constrain the generalizability of our findings. Secondly, the study primarily relied on self-reported data from participants, which may introduce recall bias, particularly when assessing subjective experiences such as loneliness, depression, and internet addiction. Although self-reported data provide a convenient means of data collection, unverified responses may be influenced by participants’ current psychological states and the accuracy of their recollections. Lastly, the study adopted a cross-sectional research design, which limits causal inferences. Although structural equation modeling allows for the exploration of correlations and potential pathways among variables, it cannot establish causal relationships. Future research could consider adopting a longitudinal design to more accurately trace the processes and causal mechanisms through which family environmental factors impact children’s and adolescents’ psychological health.

## 5. Conclusions

This study showed a high burden of mental health problems (loneliness, depression, and internet addiction) among children and adolescents in rural China. By using the modified FSM framework, this study indicated family economic conditions influence children’s and adolescents’ loneliness, depression, and internet addiction through the psychological health of primary caregivers, family communication, and hostile parenting styles. Family communication and hostile parenting styles were found to play a more significant role compared to caregivers’ psychological health, as they directly influenced loneliness, depression, and internet addiction across all models. Therefore, when designing intervention strategies for adolescents’ psychological health in rural areas, it is crucial to consider multiple dimensions of the family environment. As new issues such as internet addiction emerge, greater attention should be given to optimizing family atmosphere and parenting practices.

## Figures and Tables

**Table 1:** Sociodemographic, psychological and behavioral characteristics, and family environment of participants.

Variables	*n* (%)	Mean (SD)
**Children and adolescents’ sociodemographic characteristics**		
Gender		
Male	294 (45.1)	
Female	358 (54.9)	
Age (years)		13.06 (2.16)
Children	297 (45.6%)	
Adolescent	355 (54.4%)	
Education level		
Primary school	280 (42.9)	
Junior high school	240 (36.9)	
High school and above	132 (20.2)	
**Children and adolescents’ psychological and behavioral characteristics**		
R-UCLA score (Loneliness)		2.60 (2.84)
CES-D score (Depression)		6.42 (6.68)
CIAS-R score (Internet Addiction)		50.35 (21.55)
**Family environment**		
Primary caregiver		
Both parents	209 (32.1)	
One parent	184 (28.2)	
Grandparents	259 (39.7)	
Education level of the Primary caregiver		
Primary school or below	81 (12.4)	
Junior high school	340 (52.2)	
High school	198 (30.4)	
University and above	33 (5.0)	
Economic situation (per capita)		
<1000 CNY ^a^	21 (3.2)	
1000–2000 CNY	148 (22.7)	
2000–3000 CNY	266 (40.8)	
3000–5000 CNY	150 (23)	
>5000 CNY	67 (10.3)	
R-UCLA and CES-D score (Parents’ emotional well-being)		32.34 (8.08)
Caregivers’ expectation for children		
High	182 (27.9)	
Middle	289 (44.3)	
Low	181 (27.8)	

^a^ CNY = Chinese Yuan.

**Table 2:** Goodness-of-fit test for modified FSM model and original FSM model.

Model	*χ* ^2^	*d.f.*	*χ*^2^/*d.f.*	CFI	TLI	RMSEA	SRMR
Loneliness ^a^	786.351	340	2.31	0.970	0.967	0.045	0.031
Loneliness ^b^	391.023	147	2.66	0.981	0.978	0.050	0.027
Depression ^a^	943.302	454	2.08	0.973	0.970	0.041	0.029
Depression ^b^	511.593	226	2.26	0.982	0.980	0.044	0.026
Internet addiction ^a^	817.434	367	2.23	0.972	0.969	0.043	0.031
Internet addiction ^b^	1113.05	165	6.75	0.931	0.921	0.094	0.302

^a^ Based on modified FSM model; ^b^ based on original FSM model.

**Table 3:** The indirect effects of loneliness in the Structural Equations Model.

Pathways	β	95% CI	
		Lower	Upper
Loneliness			
a. Economic situation → Hostile parenting → Family communication → Loneliness	0.004	−0.006	0.011
b. Economic situation → Parents’ emotional well-being → Hostile parenting → Family communication → Loneliness	−0.059	−0.089	−0.030
c. Economic situation → Family communication → Loneliness	−0.013	−0.035	0.009
d. Economic situation → Expectation → Loneliness	−0.006	−0.025	0.013
e. Economic situation → Hostile parenting → Loneliness	0.002	−0.006	0.011
f. Economic situation → Parents’ emotional well-being → Loneliness	−0.075	−0.121	−0.028
g. Economic situation → Parents’ emotional well-being → Hostile parenting → Loneliness	−0.059	−0.100	−0.018
h. Economic situation → Parents’ emotional well-being → Expectation → Loneliness	−0.001	−0.002	0.001
i. Economic situation → Parents’ emotional well-being → Family communication → Loneliness	−0.076	−0.113	−0.040

**Table 4:** The indirect effects of depression in the Structural Equations Model.

Pathways	β	95% CI	
		Lower	Upper
Depression			
a. Economic situation → Hostile parenting → Family communication → Depression	0.001	−0.004	0.007
b. Economic situation → Parents’ emotional well-being → Hostile parenting → Family communication→ Depression	−0.036	−0.060	−0.012
c. Economic situation → Family communication → Depression	−0.010	−0.025	0.005
d. Economic situation → Expectation → Depression	−0.009	−0.029	0.010
e. Economic situation → Hostile parenting → Depression	0.002	−0.005	0.009
f. Economic situation → Parents’ emotional well-being → Depression	−0.132	−0.184	−0.080
g. Economic situation → Parents’ emotional well-being → Hostile parenting → Depression	−0.046	−0.087	−0.005
h. Economic situation → Parents’ emotional well-being → Expectation → Depression	−0.001	−0.003	0.001
i. Economic situation → Parents’ emotional well-being → Family communication → Depression	−0.046	−0.076	−0.016

**Table 5:** The indirect effects of internet addiction in the Structural Equations Model.

Pathways	β	95% CI	
		Lower	Upper
Internet addiction			
a. Economic situation → Hostile parenting → Family communication → Internet addiction	0.015	−0.045	0.076
b. Economic situation → Parents’ emotional well-being → Hostile parenting → Family communication → Internet addiction	−0.418	−0.630	−0.206
c. Economic situation → Family communication → Internet addiction	−0.137	−0.297	0.022
d. Economic situation → Expectation → Internet addiction	0.097	−0.045	0.238
e. Economic situation → Hostile parenting → Internet addiction	0.016	−0.048	0.080
f Economic situation → Parents’ emotional well-being → Internet addiction	0.110	−0.224	0.443
g. Economic situation → Parents’ emotional well-being → Hostile parenting → Internet addiction	−0.439	−0.747	−0.132
h. Economic situation → Parents’ emotional well-being → Expectation → Internet addiction	0.009	−0.005	0.022
i. Economic situation → Parents’ emotional well-being → Family communication → Internet addiction	−0.529	−0.795	−0.262

## Data Availability

The raw data supporting the conclusions of this article are not publicly available due to ethical review restrictions and participant confidentiality requirements but can be provided by the corresponding authors upon request.

## Appendix A

**Table A1:** Correlations among all constructions.

Variables	1	2	3	4	5	6	7	8	9
1. Loneliness	1								
2. Depression	0.897 **	1							
3. Internet Addiction	0.590 **	0.531 **	1						
4. Economic Condition	−0.273 **	−0.255 **	−0.232 **	1					
5. Family Communication	−0.768 **	−0.721 **	−0.661 **	0.328 **	1				
6. Expectation	−0.269 **	−0.265 **	−0.175 **	0.525 **	0.262 **	1			
7. Primary caregivers’ emotional well-being	0.802 **	0.790 **	0.648 **	−0.370 **	−0.787 **	−0.285 **	1		
8. Warm Parenting	−0.773 **	−0.761 **	−0.651 **	0.301 **	0.776 **	0.270 **	−0.829 **	1	
9. Hostile Parenting	0.786 **	0.754 **	0.669 **	−0.311 **	−0.765 **	−0.266 **	0.837 **	−0.854 **	1

Note: ** Correlation is significant at the 0.01 level (2-tailed).
